# Dimensionality reduction and metabolite panel derivation in urinary metabolomics based on Random Forest with Gini index feature selection

**DOI:** 10.1007/s00216-026-06759-w

**Published:** 2026-09-02

**Authors:** Markus Zetes, Vlad Moisoiu, Carmen Socaciu, Nicolae Leopold

**Affiliations:** 1 Faculty of Physics, Babeș-Bolyai University, 400084 Cluj-Napoca, Romania; 2 Faculty of Food Science and Technology, University of Agricultural Sciences and Veterinary Medicine, 400372 Cluj-Napoca, Romania; 3 BIODIATECH Research Centre for Applied Biotechnology in Diagnosis and Molecular Therapy, SC Proplanta SRL, 400478 Cluj-Napoca, Romania

**Keywords:** Metabolomics, Random Forest, Gini index, MetaboAnalyst, Urine, Breast cancer

## Abstract

Untargeted urinary metabolomics represents a promising approach for investigating metabolic alterations associated with oncogenic processes such as breast cancer (BC). However, the stable selection of informative m/z features remains a central challenge in biomarker-oriented studies, particularly in the context of early BC detection and population-level screening. Urine samples from two independent cohorts (*n* = 50 and *n* = 75) and analyzed using distinct UHPLC–QTOF–ESI⁺ mass spectrometry workflows, yielded 224 and 129 aligned m/z features, respectively. Classification and embedded feature selection were implemented within a leakage-controlled Random Forest (RF) framework using Gini index-based importance ranking. Model evaluation incorporated repeated train-test splits and cross-validation to ensure methodological rigor and minimize overfitting. By consistently applying the same RF-based analytical framework to two analytically distinct cohorts generated under different chromatographic separation conditions, we demonstrate that a unified supervised strategy can achieve comparably high classification performance despite differences in feature dimensionality. Further, controlled dimensionality reduction identified compact panels of 25 m/z features per cohort while preserving classification performance. Importantly, stability was maintained after feature reduction, with strong accuracy, F1 scores, and receiver operating characteristic and precision–recall characteristics observed in both full and reduced models. This cross-cohort consistency indicates that the discriminative signal captured by the RF approach is not cohort-specific nor dependent on a particular separation workflow, but rather reflects reproducible metabolic patterns associated with BC. The stability of feature selection was further supported by substantial overlap between RF-derived Gini importance rankings and variable importance in projection (VIP) scores obtained from partial least squares discriminant analysis (PLS-DA) in MetaboAnalyst 5.0, indicating concordance across distinct supervised multivariate frameworks. Collectively, these findings highlight the advantage of a unified, supervised tree-based strategy capable of delivering stable classification and interpretable dimensionality reduction across independent untargeted metabolomics platforms, providing a structured and transferable framework for metabolomics-driven biomarker discovery and future clinical validation.

**Supplementary Information:**

The online version contains supplementary material available at https://doi.org/10.1007/s00216-026-06759-w.

## Introduction

Despite substantial advances in imaging and molecular diagnostics for breast cancer (BC), metabolic biomarkers reflecting perturbations associated with oncogenic processes remain of considerable interest, particularly for early BC detection and large-scale population screening [1–3]. Untargeted liquid chromatography–mass spectrometry (LC–MS/MS) enables comprehensive characterization of metabolic alterations linked to tumor biology, providing a broad and unbiased view of cancer-associated metabolic reprogramming [4].

Metabolomics has emerged as a powerful analytical approach for investigating disease-associated metabolic alterations and identifying clinically relevant biomarkers, playing an increasingly important role in precision medicine [5]. Advanced analytical platforms such as nuclear magnetic resonance (NMR) spectroscopy and mass spectrometry (MS) enable comprehensive profiling of metabolites in complex biological matrices, including serum and urine, and have demonstrated the ability to discriminate cancer patients from healthy controls based on distinct metabolic signatures [6]. In oncology, metabolomics is particularly valuable for detecting low-molecular-weight biomarkers, such as modified nucleosides, which reflect tumor-related metabolic reprogramming [7]. However, the high dimensionality and complexity of metabolomics datasets require the application of chemometric and machine learning approaches for reliable interpretation. Multivariate statistical methods and advanced data processing strategies, including multi-platform data fusion, have therefore become essential for classification, feature selection, and biomarker discovery, ultimately enhancing the robustness and clinical applicability of metabolomics-based cancer diagnostics [8].

Urine represents an attractive biofluid for metabolomic investigations due to its non-invasive collection and its ability to integrate metabolic activity across multiple physiological pathways. LC–MS-based studies have reported consistent alterations in amino acid, lipid, nucleoside, and steroid metabolism in BC patients, underscoring the potential of metabolomic profiling for biomarker discovery and disease stratification [9–11]. However, the principal challenge in untargeted metabolomics lies not in data acquisition but in data interpretation. High-dimensional datasets, in which the number of detected variables approaches or exceeds the number of samples, increase the risk of model overfitting, limit reproducibility, and complicate downstream biological validation.

Multivariate analysis techniques, and more recently machine learning algorithms, have significantly enhanced the analytical power of metabolomics by facilitating the interpretation of complex datasets and revealing patterns not readily detectable using conventional statistical methods. Platforms such as MetaboAnalyst 5.0 [12] provide widely used tools for metabolomic data analysis, including projection-based approaches such as Principal Component Analysis (PCA) and Partial Least Squares Discriminant Analysis (PLS-DA), which have proven effective in distinguishing between BC stages and identifying predictive biomarkers [13, 14]. However, although powerful for pattern recognition, these projection-based methods transform original m/z features into latent variables, which may reduce direct biochemical interpretability.

Supervised machine learning offers an alternative analytical framework that preserves feature identity while enabling robust classification. In this study, we implemented Random Forest (RF) modeling with embedded Gini index–based feature ranking for controlled dimensionality reduction. Unlike projection-based techniques, RF retains individual metabolite identity while capturing non-linear relationships and feature interactions, thereby enabling interpretable prioritization of discriminative m/z features within the original feature space.

RF is particularly well suited for metabolomics-based cancer classification due to its strong performance in small patient cohorts and its capacity to handle high-dimensional datasets [15]. In metabolomics studies, where the number of measured variables frequently exceeds the number of available samples [16], this property is especially important. One of the principal advantages of RF is its relative resistance to overfitting, making it appropriate for complex clinical datasets characterized by limited sample size and high dimensionality.

A further strength of RF lies in its embedded feature selection capability. By leveraging importance metrics such as Mean Decrease Accuracy (MDA) and Mean Decrease Gini (MDG), the algorithm ranks variables according to their contribution to classification performance, thereby enabling meaningful and controlled dimensionality reduction. The Gini index, a statistical measure used in decision tree–based models, quantifies how effectively a given feature improves class separation by increasing node purity [10]. Although alternative splitting criteria such as entropy and information gain are used in other tree-based algorithms, the Gini index offers computational efficiency and comparable performance, making it particularly suitable for high-dimensional metabolomics datasets.

The present study was motivated by two objectives: (i) to evaluate the discriminative capacity of untargeted urinary metabolomic profiles for BC classification using a leakage-aware supervised learning strategy consistently applied across two independent cohorts generated under different chromatographic separation conditions, and (ii) to establish a reproducible and interpretable dimensionality reduction approach that preserves biochemical meaning while maintaining classification performance across analytically distinct datasets.

Untargeted urinary metabolomics was applied to two independent patient cohorts [13, 14], comprising 224 and 129 detected m/z features, respectively, obtained using distinct high-resolution LC–MS workflows. By applying the same RF-based analytical framework to both cohorts, we were able to directly assess methodological robustness across different separation conditions and feature spaces. Using RF-based classification with Gini index–driven feature selection, we identified concise panels of 25 highly informative m/z features per cohort that accurately discriminated BC patients from healthy controls.

To further strengthen the validity of feature selection, Gini-derived m/z feature rankings were compared with Variable Importance in Projection (VIP) scores obtained from PLS-DA analysis performed in MetaboAnalyst 5.0 [12]. The observed overlap between methods highlighted a subset of robust and consistently prioritized m/z features. Importantly, high classification accuracy was maintained even after reduction to the 25-m/z feature panels, supporting the feasibility of integrating supervised machine learning with untargeted metabolomics for reliable, non-invasive BC classification. RF-Gini feature selection is not presented here as an entirely new algorithmic approach for metabolomic applications. Rather, the novelty of the present work lies in its structured application as a leakage-aware, feature-preserving dimensionality reduction strategy and in its direct comparison with PLS-DA VIP scores and MetaboAnalyst RF rankings across two urinary metabolomics datasets generated under different UHPLC-QTOF-MS conditions.

Collectively, this work provides a structured, leakage-aware, and feature-preserving analytical framework that enhances interpretability while maintaining predictive performance across independent chromatographic workflows.

## Materials and methods

### Urine collection and UHPLC–QTOF-ESI^+^ MS analysis

Urine samples were obtained from two previously generated urinary metabolomics cohorts comprising breast cancer (BC) patients and healthy female controls (Ctrls). Cohort A included 50 participants (35 BC, 15 Ctrl), while cohort B included 75 participants (53 BC, 22 Ctrl). The two datasets originated from separate experimental studies and were analyzed independently in the present work; no model was trained on one cohort and externally tested on the other. Based on the available published cohort descriptions, cohort A showed an age difference between BC and Ctrl participants (55.6 ± 11.1 years vs. 44.6 ± 5.2 years), while BMI and menopausal distribution were more similar between groups. Because individual-level demographic data were not available for all participants in the present secondary analysis, formal age-adjusted machine learning models could not be performed. Therefore, age-related effects cannot be excluded and this is acknowledged as a limitation of the study. However, the primary aim of the present work was not to establish a clinically validated diagnostic putative discriminatory m/z feature panel, but to compare feature-ranking strategies across real urinary metabolomics datasets [17, 18]. All participants were hospitalized for at least one week prior to sample collection and received standardized meals, which minimized dietary variability. The diagnosis of BC was confirmed via tissue biopsy in accordance with the AJCC Cancer Staging Manual (8th Edition). The study was conducted in strict adherence to ethical guidelines approved by the institutional ethics committee, and in accordance with the Declaration of Helsinki and its subsequent amendments.

Urine samples were collected from both groups of participants (BC and Ctrl), in the morning, following a minimum 12-h fasting period, to minimize diurnal and dietary variations. For BC patients, samples were obtained prior to any surgical intervention. Upon collection in sterile containers, samples were centrifuged (1500 rpm at 4 °C) to remove cellular debris, then aliquoted and stored at −80 °C until analysis. For analysis, thawed urine samples from both cohorts were mixed with the extraction solvents described in Table 1 and incubated at − 20 °C for 24 h. After a second centrifugation at 12,500 rpm for 10 min, the supernatant was filtered through PTFE microfilters (0.25 μm) and introduced in the autosampler. Aliquots of 5 μl supernatant were subjected to analysis on a Thermo Scientific UHPLC DionexUltiMate 3000 system equipped with a quaternary pump system and a photodiode array detector and coupled with a Quadrupole Time‐of‐Flight Mass Spectrometer (QTOF-ESI⁺ MS), in a mass range of 50–1000 m*/z*, with periodic calibration and quality control injections implemented to guarantee data consistency and reproducibility throughout the analytical run. Quality control (QC) procedures were implemented to ensure analytical stability. Mass calibration was performed prior to each analytical batch, maintaining mass accuracy at approximately 10 ppm throughout the runs. QC samples consisting of pooled urine from all study groups were analyzed and used for feature filtering and alignment.

**Table 1:** Data regarding the protocols for extraction, processing and metabolomic analysis of urine samples from cohorts A and B

	Cohort A	Cohort B
Urine samples (n)	50 (15 Ctrl + 35 BC)	75 (22 Ctrl + 53 BC)
Solvents for extraction (per 1 ml urine)	1.5 ml (MeOH: AcN, 1:1)	4 ml MeOH
UHPLC column/flow	Acclaim 3 µm (2.1 × 100 mm) at 40 °C; Flow of 0.5 ml/min. Universally used for polar and non-polar molecules separation	Acquity ACQUITY UPLC BEH C18 3 µm (2.1 × 50 mm) at 30 °C; Flow of 0.8 ml/min. More suited for nonpolar to moderately polar compounds
Elution solvents and gradient program	*Solvent A*: H_2_O with 0.1% formic acid; *Solvent B*: AcN with 0.1% formic acid; Min 0: 5%; Min 3: 15%; Min 6: 50%; Min 9: 95%; Min 17: 5%	
Retention time range	2–16 min	6–11 min
Aligned common molecules in BC vs Ctrl	*n* = 224	*n* = 129
Mass range (*m/z*)	104–900	111–600

Feature extraction was carried out using the Find Molecular Features (FMF) and Extracted Ion Chromatogram (EIC) algorithms (Data Analysis 4.2, Bruker Daltonics), generating datasets including retention time, *m/z*, peak intensity, peak area, and signal-to-noise ratio. Only features fulfilling the following criteria were retained: intensity > 5000, S/N > 5, presence in at least 50% of QC samples, and *m/z* < 900 Da.

Further alignment and filtering were performed using the NEAPOLIS platform, retaining features present in more than 60% of samples. These steps ensured data consistency and reduced technical variability prior to statistical analysis.

Two independent untargeted metabolomic datasets were generated from the UHPLC–QTOF-ESI⁺ MS analysis of urine samples from cohorts A and B. Cohort A (*n* = 50), yielded 224 m/z features shared between Ctrl and BC groups, while cohort B (*n* = 75) yielded 129 shared features, as reported previously [17, 18].

Raw mass spectrometric data were processed using a standardized workflow comprising automated peak detection, deconvolution, and retention time alignment. Data Analysis 4.2 (Bruker Daltonics) was applied to individual total ion chromatograms, and the “Find Molecular Features” algorithm was used to generate feature matrices containing retention time, peak area, peak and intensity, signal-to-noise (S/N) ratio, and corresponding *m/z* [M + 1] values for each detected component. Chromatographic peaks with retention times under 1 min, intensities below 3000 units, S/N values below 3, and *m/z* values above 900 Daltons were excluded from further analysis. Throughout the manuscript, the term m/z features refers to LC–MS signals retained after preprocessing and filtering. Where database matching was performed, these are described as putatively annotated m/z features; because MS/MS spectra and authentic standards were not available, they should not be interpreted as confirmed metabolite identifications.

### Statistical and machine learning analysis

For comparative statistical analysis, MetaboAnalyst 5.0 [12] a widely used web-based platform for metabolomics data processing and visualization was employed. The platform provides a comprehensive suite of multivariate tools, including Volcano plot visualization, Principal Component Analysis (PCA), Partial Least Squares Discriminant Analysis (PLS-DA; score plots and Variable Importance in Projection (VIP) scores), Random Forest (RF)-based classification and feature selection, correlation analysis between samples and variables, and heatmap construction for visualization of these correlations.

Supervised classification was performed using a RF algorithm implemented in Python. RF hyperparameters were fixed a priori to avoid data-driven optimization and potential information leakage. The model configuration included 200–400 trees, a maximum tree depth of 6 a minimum of 5–10 samples required for node splitting, a minimum of 2–8 samples per terminal node, and the square root of the total number of features considered at each split. For cohort B, class weights were adjusted to account for class imbalance.

Model performance was evaluated using a leakage-safe validation strategy. For visualizations, six independent outer train–test splits were generated using fixed random seeds. In each split, 80% of the samples were used for training and 20% were held out as an independent test set. All preprocessing steps, including L2 normalization and feature ranking, were performed exclusively on the training data within each split and subsequently applied to the corresponding test data.

Feature importance was quantified using the Gini index (Mean Decrease Gini) intrinsic to the RF model. Gini importance values were computed on training data only and averaged across all outer splits to generate a stable ranking of discriminative m/z features. Based on this ranking, a reduced m/z feature panel (top 25 features) was defined and evaluated separately to assess the impact of dimensionality reduction on classification performance. Because the two cohorts differed in sample preparation and UHPLC-QTOF-MS acquisition parameters, they were not merged into a single harmonized dataset. No cross-cohort normalization, batch correction, or pooled scaling was applied. Instead, preprocessing, normalization, filtering, feature selection, and RF modeling were performed independently within each cohort.

Classification performance was quantified using accuracy, recall, F1 score, receiver operating characteristic area under the curve (ROC-AUC), and precision–recall area under the curve (PR-AUC). For the six outer splits, metrics were reported per split and summarized as mean ± standard deviation. Confusion matrices were generated for each test split.

To obtain more robust and less optimistic performance estimates, an additional evaluation was conducted using Repeated Stratified K-Fold cross-validation, aggregating predictions across multiple folds and repetitions. This procedure enabled stable estimation of performance metrics and construction of mean ROC and PR curves across repeated test predictions. Training and test performance were reported separately to assess potential overfitting.

To validate feature selection stability, RF-derived Gini rankings were compared with VIP scores obtained from PLS-DA analysis in MetaboAnalyst 5.0. Concordance between the two methods was used to identify consistently informative m/z features.

All computational analyses were performed using the Anaconda distribution with Python version 3.9.15 as the primary programming environment. Machine learning analyses were implemented using the scikit-learn library, while pandas and NumPy were employed for data handling and numerical computation. Data visualization was performed using matplotlib and seaborn in Python.

All machine learning workflows were executed within a Jupyter Notebook environment, enabling transparent, stepwise documentation of preprocessing, model training, validation, and visualization. Fixed random seeds were used where applicable to ensure reproducibility. Model evaluation procedures, including train–test splitting, normalization, feature ranking, and metric computation, were implemented explicitly within each validation fold to prevent information leakage.

Statistical visualization and supplementary exploratory analyses were additionally performed using MetaboAnalyst 5.0 and R-based plotting tools where indicated. This combined computational framework ensured a reproducible, transparent, and analytically rigorous pipeline for metabolomic data analysis and machine learning-based classification.

## Results and discussion

Gini-based Random Forest (RF) models applied to two untargeted metabolomics cohorts achieved high classification accuracy in distinguishing breast cancer (BC) from control (Ctrl) urine samples. The analysis employed rigorous validation strategies, including multiple randomized splits and cross-validation procedures, to ensure robustness and generalizability of the predictive models. Importantly, the classification performance was confirmed through independent validation, with the trained models tested on an independent dataset to evaluate their reproducibility and real-world applicability.

### Classification performance of RF machine learning model

The cohort A dataset comprised 224 urinary m/z features determined from urine samples of 35 BC patients and 15 Ctrl subjects (see “Urine collection and UHPLC–QTOF-ESI+ MS analysis”). Classification performance was evaluated using a leakage-safe RF framework with repeated train-test splitting. In each split, 80% of the samples were used for training and 20% were held out as an independent test set (see “Statistical and machine learning analysis”).

**Fig. 1:**
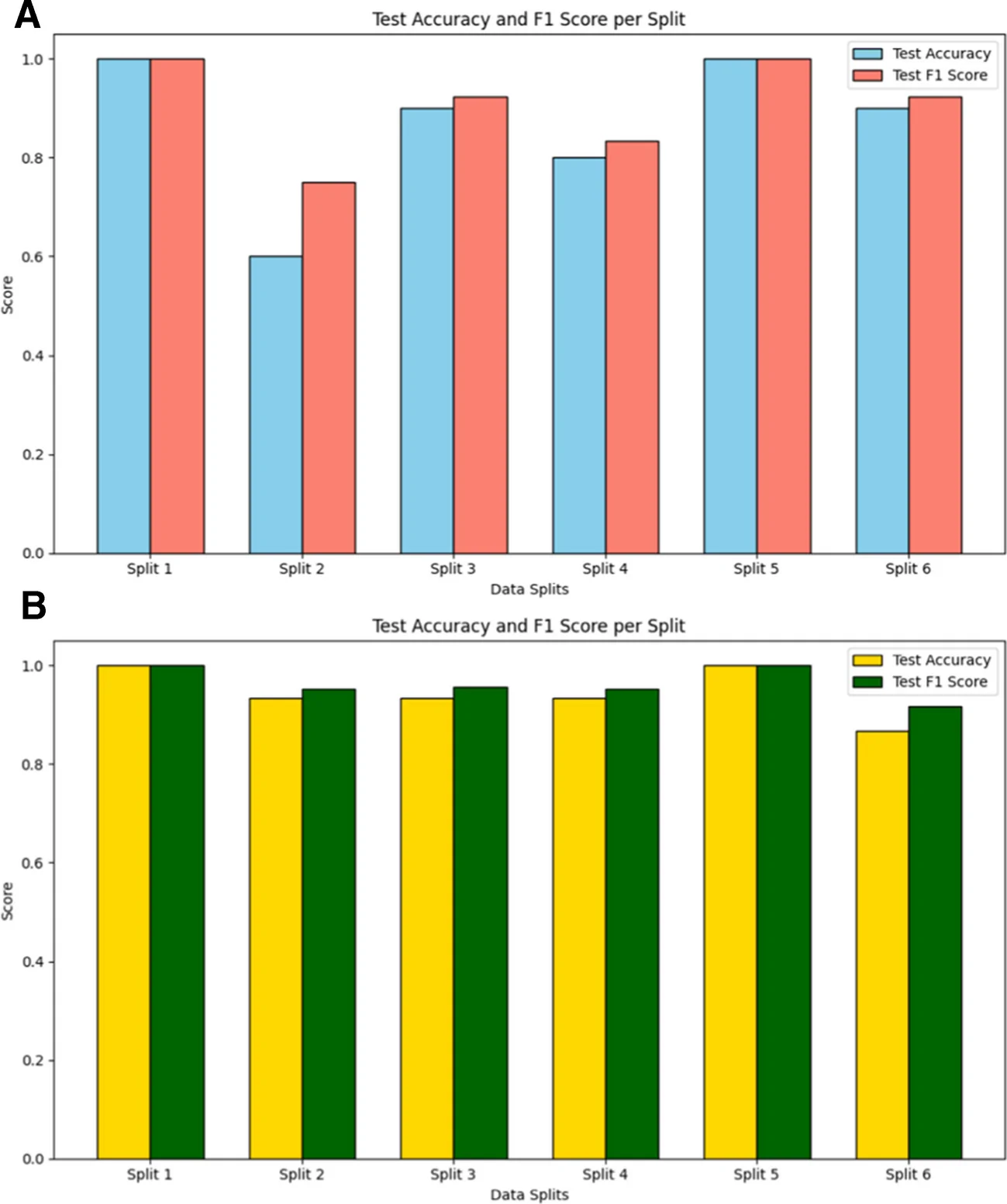
Classification performance with independent validation data, based on the complete set of m/z features. Test set accuracy and F1 score are shown across six independent test splits using Random Forest (RF) with Gini index-based feature selection for (**A**) cohort A, comprising 224 urinary m/z features, and (**B**) cohort B, comprising 129 urinary m/z features

Across six independent training outer splits, the RF model achieved a mean training accuracy of 91.7% (with standard deviation (SD) = 0.03) and a mean training F1 score of 94.0% (SD = 0.02). On the corresponding independent test sets, the mean accuracy was 86.7% (SD = 0.15) and the mean F1 score reached 90.5% (SD = 0.09), with performance variability across splits (60%–99%) reflecting the limited cohort size and class imbalance. Importantly, when aggregated across all splits, overall performance remained consistently high, indicating stable learning behavior. Figure 1A presents test-set accuracy and F1 scores across six independent splits. The classification results indicate model performance with the bar charts showing accuracy and F1 scores that closely align between cross-validation on the training set and independent testing conditions.

In contrast to cohort A, which shows higher inter-split variability, cohort B demonstrates consistently high and stable performance (Fig. 1B), in line with its larger sample size (53 BC patients and 22 Ctrls). Based on the 129 urinary m/z features, the RF model achieved a mean training accuracy of 98.6% (SD = 0.01) and training F1 score of 99.0% (SD = 0.01), while independent test sets yielded a mean accuracy of 94.4% (SD = 0.05) and mean F1 score of 96.3% (SD = 0.03).

Receiver operating characteristic (ROC) curves were generated from repeated held-out test predictions for both cohorts and are provided in the Supplementary Information (Figure S4). In cohort A, the mean ROC-AUC was 0.96 ± 0.06, while cohort B showed near-perfect discrimination, with mean ROC-AUC approximately 1.00 across repeated test splits. Because each held-out test split contained a limited number of samples, the ROC curves display the expected step-like behavior of small test partitions. Accordingly, the ROC curves are interpreted together with the repeated-split performance metrics and should not be considered evidence of external clinical validation.

Although cohort B yielded ROC-AUC and PR-AUC values close to unity, these results should be interpreted with caution. The high values may reflect strong class separation within this dataset, but they may also be influenced by the moderate sample size, cohort-specific structure, analytical workflow, class composition, or residual demographic confounding. Therefore, the near-perfect AUC values should not be interpreted as evidence of clinical-grade diagnostic performance. Rather, they indicate that the selected feature-ranking and classification workflow captured a strong discriminatory signal within cohort B. Confirmation in larger, prospectively collected, demographically balanced, and externally validated cohorts will be required before any diagnostic or translational conclusions can be drawn.

**Fig. 2:**
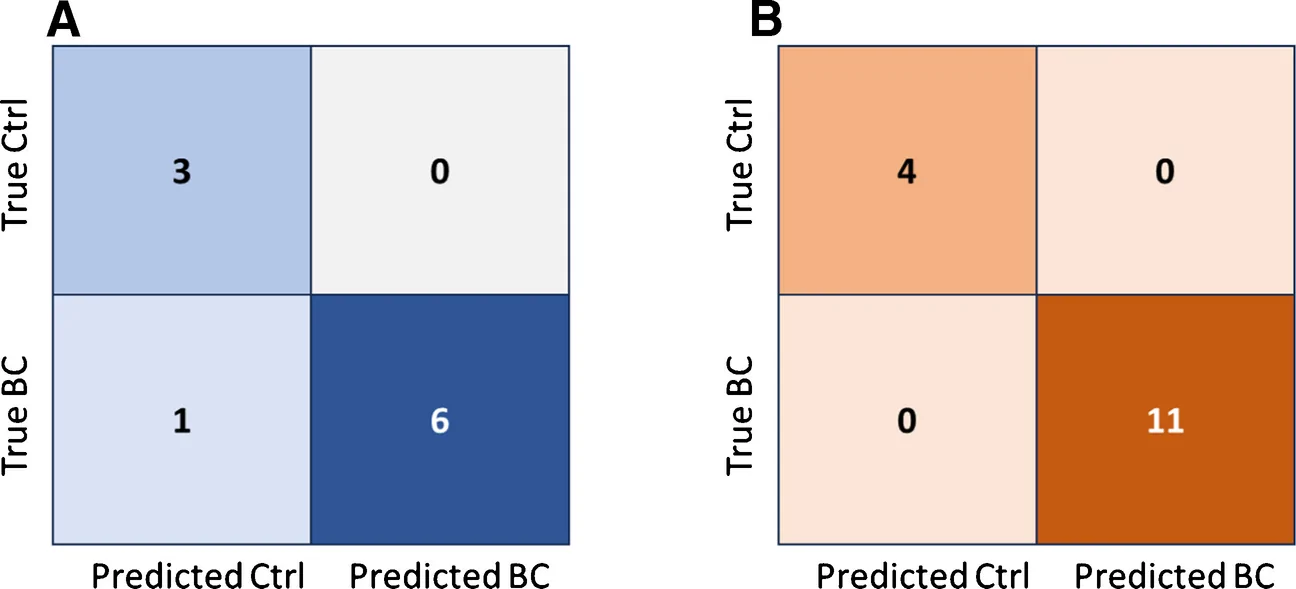
Confusion matrices illustrating test set mean classification performance using Random Forest (RF) with Gini index-based feature selection for (**A**) cohort A and (**B**) cohort B, based on the complete set of m/z features

Classification behavior is further illustrated by the confusion matrices shown in Fig. 2, summarizing performance on the independent test sets (Fig. 1). Mean confusion matrices across the six splits are presented in Fig. 2, while split-specific matrices are provided in Figures S1 and S2 (Supplementary Information).

Both cohorts display a pronounced diagonal structure in their mean confusion matrices, reflecting high true positive and true negative rates, indicative of reliable model performance. Misclassifications were limited and primarily observed in cohort A, consistent with its smaller sample size and higher variance across splits. Collectively, these results indicate that RF-based modeling of urinary metabolomic data yields robust and reproducible classification performance, supporting its suitability for metabolomics-driven biomarker identification in BC.

### Controlled dimensionality reduction

To determine whether robust classification could be achieved with a reduced and meaningful feature set, we implemented a controlled dimensionality reduction strategy based on RF Gini index feature importance. In untargeted metabolomics, the number of detected variables often surpasses the number of samples, increasing the risk of overfitting and limiting interpretability. Feature selection therefore represents not only a statistical necessity but also a strategic step toward defining a discriminative metabolite feature panel with potential clinical relevance. Rather than relying on unsupervised compression techniques that recast the data into latent variables detached from the original m/z features, we leveraged the embedded feature-ranking capability of RF to preserve feature identity and prioritize those most informative for BC classification.

Within each training split, feature importance was quantified using the Mean Decrease Gini (MDG) metric, which reflects the average reduction in node impurity contributed by a feature across all trees in the ensemble. The Gini index measures the impurity reduction achieved by splitting nodes in decision trees, thus quantifying each feature’s contribution to the model’s predictive performance [14]. Specifically, a higher Gini index value indicates that a particular metabolite feature more effectively separates classes, enhancing node purity and classification accuracy.

Within each training split, feature importance was quantified using the MDG metric, which reflects the average reduction in node impurity contributed by a feature across all trees in the ensemble. The Gini index measures how effectively a given feature improves class separation by increasing node purity in decision tree–based models. Importantly, Gini importance values were computed exclusively on training data and subsequently averaged across all six outer splits to obtain a stable, leakage-free ranking of m/z features. Higher Gini index values indicate a stronger contribution to the model’s discriminative performance.

**Fig. 3:**
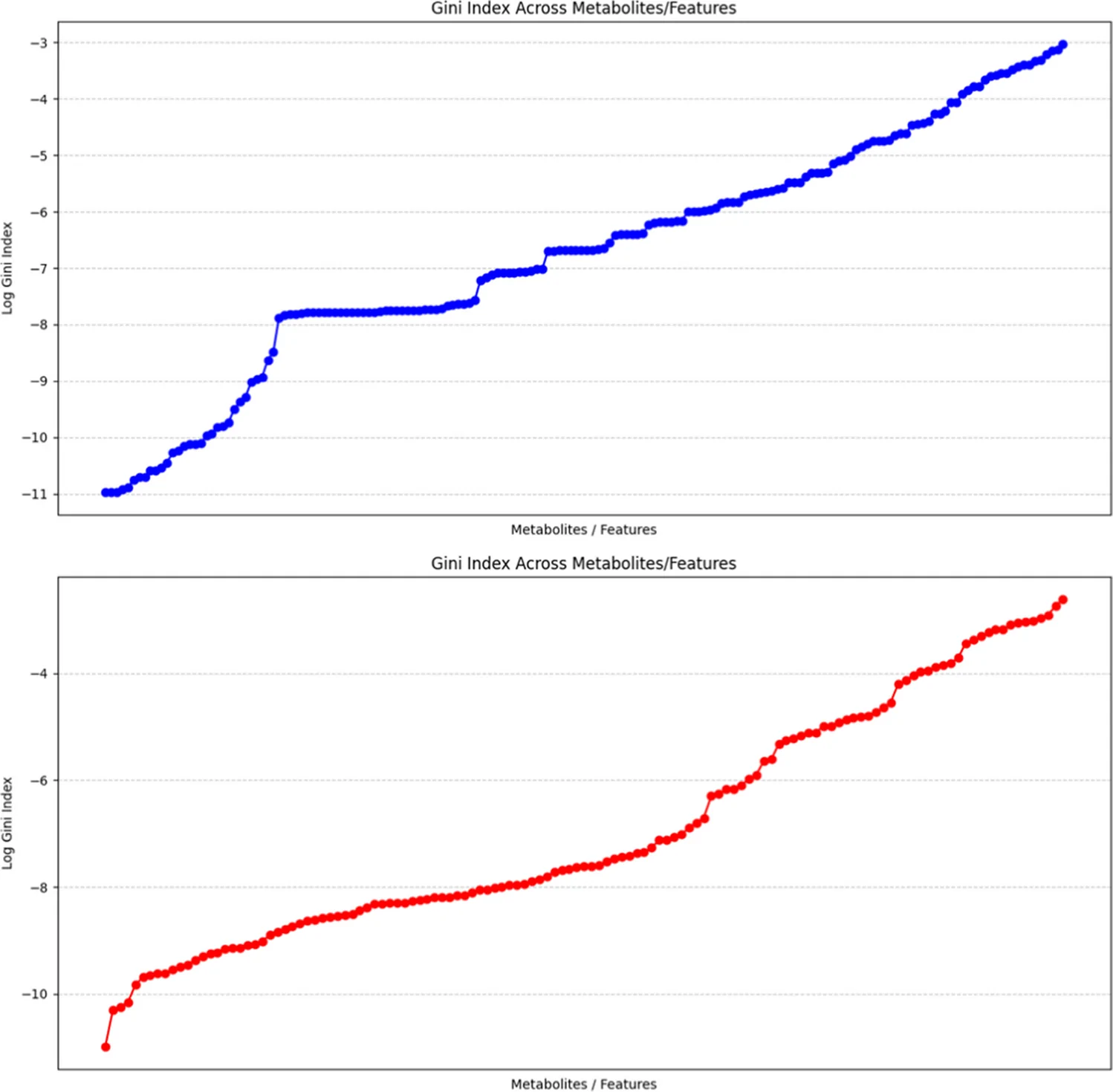
m/z feature importance ranking for (**A**) cohort A and (**B**) cohort B, calculated using the mean Gini index obtained from Random Forest (RF) classification across six independent data splits. Features positioned in the upper right corner of each panel represent the most influential m/z features

Figure 3 presents the distribution of m/z feature importance scores for cohorts A and B, displayed as the natural logarithm of the mean Gini index averaged across splits. m/z features are ordered by ascending importance along the x-axis, while higher (less negative) log-Gini values on the y-axis correspond to stronger discriminative power. This visualization highlights a gradual decline in feature importance rather than a sharp cutoff, supporting the rationale for selecting a limited subset of top-ranked features.

Based on this ranking, a reduced panel of 25 highly informative m/z features was defined for each cohort. The identities and importance rankings of these features are reported in Table S1 (Supplementary Information), while Table 2 summarizes the top 25 features for cohorts A and B and compares RF-derived Gini rankings with VIP scores obtained from PLS-DA analysis in MetaboAnalyst 5.0. Features consistently ranked highly by both methods can be considered high-confidence candidates for classification.

Notably, when the RF classifier was retrained using only the top 25 features, classification performance remained comparable to that obtained with the full feature set in both cohorts. In cohort A, mean test accuracy and F1 score were preserved within the variability observed across splits, while in cohort B, reduced-panel performance closely matched that of the full model. These findings indicate that a substantial proportion of discriminatory information is captured by a relatively small subset of m/z features.

The use of Gini index–based feature selection therefore provides a practical and interpretable approach for dimensionality reduction in untargeted metabolomics. By narrowing the focus to a concise panel of discriminative m/z features this strategy enhances model interpretability and feasibility for downstream validation. However, given the untargeted nature of the data and the current level of metabolite annotation, the identified features should be regarded as putative biomarkers.

**Table 2:** m/z features (*n* = 25) ranked by their contribution to model performance, as quantified by the Gini index–based Random Forest (RF) model and Variable Importance in Projection (VIP) scores from partial least squares discriminant analysis (PLS-DA) in MetaboAnalyst 5.0, for cohorts A and B. m/z features common to both rankings within each cohort are highlighted in bold

Importance rank	Cohort A		Cohort B	
	*m/z* feature—Gini index	*m/z* feature—VIP score	*m/z* feature—Gini index	*m/z* feature—VIP score
1	**457.319**	**457.319**	**360.326**	**338.344**
2	**391.333**	**391.333**	343.299	**360.326**
3	644.506	**539.361**	**371.33**	**557.372**
4	**539.361**	**369.351**	**557.372**	**406.332**
5	688.533	**427.393**	129.128	**348.255**
6	161.971	**851.491**	**338.344**	**304.26**
7	184.987	**341.319**	228.27	540.451
8	**369.351**	230.251	**240.234**	280.265
9	540.456	440.362	199.171	277.186
10	**143.96**	663.466	**406.332**	545.407
11	584.484	282.282	295.194	**350.269**
12	457.356	**426.472**	239.163	496.425
13	501.383	327.081	353.268	501.38
14	135.118	612.175	**304.26**	**393.312**
15	149.134	412.456	**517.378**	**371.33**
16	**851.491**	256.302	230.25	285.28
17	**427.393**	221.155	**348.255**	256.265
18	172.956	379.285	**393.312**	299.164
19	413.328	317.137	173.152	**240.234**
20	496.428	200.239	**531.261**	**517.378**
21	**145.956**	**143.96**	200.222	457.354
22	**341.319**	228.271	214.255	365.196
23	207.111	**145.956**	288.274	**531.261**
24	429.323	897.656	**350.269**	282.281
25	**426.472**	459.493	271.19	227.166

To provide additional biochemical context for the most discriminative features identified by the machine learning models, we performed putative metabolite annotation of the top-ranked *m/z* signals detected in both cohorts. Metabolite assignment was conducted based on accurate mass matching of the experimentally observed precursor ions (assuming the [M+H]⁺ adduct) with theoretical masses from publicly available metabolomics databases, primarily the Human Metabolome Database (HMDB) and LipidMaps. A mass tolerance of 0.05 Da was applied, and the observed mass errors were within 10–50 ppm for all annotated features. Because MS/MS fragmentation data were not acquired in this study, these assignments should be regarded as putative annotations based on accurate mass matching rather than confirmed metabolite identifications. The resulting annotations for the most discriminative features are provided in the Supplementary Information for cohort A (Table S3) and cohort B (Table S4). Several of the annotated features correspond to metabolites previously reported in cancer-related metabolomic studies, including bile acid derivatives, steroid conjugates, and lipid species, supporting the biological plausibility of the selected feature panels.

The two cohorts differed in several analytical parameters, including extraction solvent, LC column, flow rate, retention time window, and effective m/z coverage. These differences may influence metabolite extraction efficiency, chromatographic separation, ionization behavior, feature detection, and the final retained feature space. Consequently, the selected m/z feature panels should not be interpreted as directly interchangeable between cohorts. As described in the “Materials and methods” section (“Statistical and machine learning analysis”), no cross-cohort harmonization, batch correction, or pooled normalization across both datasets was applied because the cohorts were not merged into a single dataset. Instead, preprocessing and normalization were performed within each cohort, and the RF-Gini workflow was applied independently to each dataset. Therefore, the comparison primarily evaluates the methodological robustness of the feature-ranking strategy across analytically distinct datasets, rather than direct reproducibility of an identical putative discriminatory m/z feature panel across platforms.

### Impact of feature reduction on model performance

To explicitly assess the effect of dimensionality reduction on classification performance, RF models were retrained using only the 25 m/z feature panels identified by Gini index–based feature ranking. Performance was evaluated using the same leakage-safe validation strategy described above, including six independent outer train–test splits and repeated stratified cross-validation.

Using the fixed 25-m/z feature panels, both cohorts maintained high classification performance that was comparable to the corresponding full-feature models. In cohort A, the reduced model achieved a mean test accuracy of approximately 90% (SD = 0.06) and a mean test F1 score of approximately 92% (SD = 0.05) across the six independent splits. More conservative evaluation using repeated stratified cross-validation yielded a mean test accuracy of approximately 86% (SD = 0.1) and a mean test F1 score of approximately 88% (SD = 0.09), together with mean ROC-AUC and PR-AUC values of approximately 0.98 (SD = 0.05) and 0.99 (SD = 0.02), respectively. These results indicate stable generalization despite reduced dimensionality and highlight the impact of limited sample size on split-to-split variability.

In cohort B, performance of the 25-metabolite feature panel was consistently stronger, due to higher overall sample number, which favors more accurate classifications Across the six independent splits, the reduced model achieved a mean test accuracy of approximately 93% (SD = 0.05) and a mean test F1 score of approximately 96% (SD = 0.04). Repeated cross-validation further confirmed these findings, yielding a mean test accuracy exceeding 97% (SD = 0.03) and a mean test F1 score of approximately 98% (SD = 0.02), together with near-perfect mean ROC-AUC and PR-AUC values of approximately 0.99 (SD = 0.01) for both metrics. As mentioned previously, these results likely reflect a clearer metabolic feature separation between classes and increased statistical stability due to the larger cohort size.

**Fig. 4:**
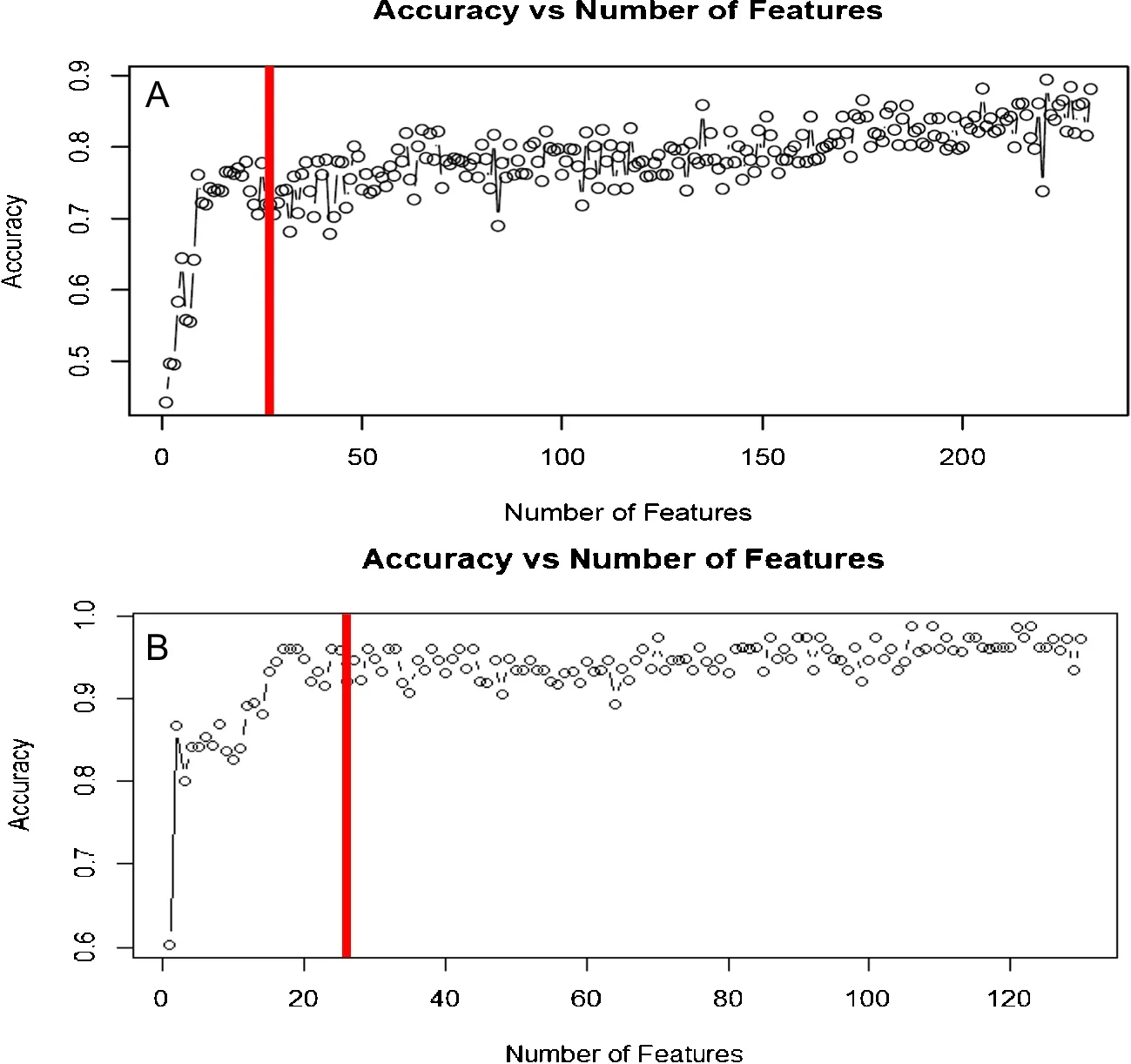
Classification accuracy as a function of the number of top-ranked m/z features selected based on the Gini index in Random Forest (RF) models. For both (**A**) cohort A and (**B**) cohort B, accuracy rapidly reached a plateau with approximately 25 features, closely matching the performance of the full model. The reduced 25-feature panels achieved testing accuracies of 80% (cohort A) and 90% (cohort B). This dimensionality reduction preserved predictive performance while improving model simplicity

To further assess the practical implications of feature importance, we analyzed how classification accuracy changed with the number of m/z features employed by the RF model. Figure 4 illustrates a striking observation: using only a small subset of top-ranked m/z features, as determined by Gini index importance, results in classification accuracies comparable to those achieved with the complete metabolite datasets.

For both cohorts, accuracy rapidly approached an optimal plateau, suggesting that adding more features beyond the top-ranked m/z features offered minimal benefit and could introduce non-informative variance (noise), potentially dilute the predictive signal and increase the risk of overfitting. Furthermore, final model evaluation was performed using only the reduced feature set. In cohort A, the test accuracy and F1 score obtained using the top 25 m/z features were 81% and 83%, respectively. In contrast, applying the same approach to cohort B yielded markedly higher performance, with a test accuracy of 95% and an F1 score of 96%.

This finding bears significant clinical and practical implications. Utilizing fewer m/z features reduces complexity, streamlines analytical processes, minimizes costs, and enhances reproducibility across independent laboratories [19]. By establishing an optimized minimal biomarker set, researchers provide clinicians with a decision-support tool that enables quicker diagnostic assays, simplifies analytical protocols, and accelerates clinical decision-making. This approach also improves patient compliance and facilitates large-scale screening by reducing resource requirements and turnaround times.

### Gini index–Variable Importance in Projection (VIP) scores alignment in m/z feature selection

Several top-ranked m/z features were consistently identified by both the Gini index–based RF approach and VIP scores derived from PLS-DA (Table 2), supporting the reliability of these complementary feature selection strategies. To assess agreement between methods, we compared m/z feature importance rankings obtained from RF (Gini index) and from PLS-DA (VIP scores) computed using the MetaboAnalyst 5.0 platform for cohorts A and B. For each method and cohort, the 25 highest-ranked m/z features were selected and compared side by side (Table 2).

Cross-comparison revealed a subset of m/z features concordantly prioritized by both methods. This agreement supports their classification as high-confidence candidates and indicates that they represent stable discriminatory signals rather than methodological artifacts.

Cross-comparison of RF-derived Gini rankings and PLS-DA VIP scores revealed substantial overlap in both cohorts. In cohort A, 10 of the 25 selected m/z features were shared between the two ranking approaches, while in cohort B, 12 of the top 25 features overlapped. In both datasets, several of these shared features were among the highest-ranked variables in each list.

This concordance between two fundamentally different multivariate frameworks—one non-linear and tree-based (RF), the other linear and projection-based (PLS-DA)—supports the reproducibility and statistical relevance of the overlapping features. While VIP scores quantify variable contribution to latent components explaining covariance between predictors and class labels, the Gini index reflects feature importance within a hierarchical decision structure that captures non-linear relationships and interactions.

The convergence of these distinct modeling strategies across two independent cohorts indicates analytical stability and suggests that the shared features represent consistent discriminatory signals rather than method-specific artifacts or cohort-dependent noise. Moreover, agreement between approaches reduces the likelihood of overfitting and strengthens confidence in these features as high-confidence candidates.

To further evaluate the stability of feature selection, an additional RF-based analysis was performed using the MetaboAnalyst 5.0 platform. Gini index rankings obtained from our Python-based RF implementation were compared with VIP scores derived from PLS-DA and with RF feature importance scores generated within MetaboAnalyst. The results of this cross-method comparison are provided in the Supplementary Information (Table S2 and Figure S3). Alternative feature-selection strategies, such as LASSO/Elastic Net regularization, Boruta, recursive feature elimination, permutation importance, and univariate filter-based methods, are also widely used in high-dimensional metabolomics datasets. Compared with these approaches, RF-Gini provides an embedded tree-based ranking that retains the original m/z feature identity and can capture non-linear relationships and feature interactions. However, because Gini importance may be influenced by correlated predictors, its results should not be interpreted in isolation. The comparison with VIP scores and MetaboAnalyst RF rankings was therefore used as a complementary strategy to assess whether similar features were prioritized by methodologically distinct approaches.

The observed concordance across implementations and modeling approaches reinforces the stability of the feature selection strategy, whereas the remaining discrepancies likely reflect methodological differences and cohort-specific variability inherent to distinct analytical frameworks.

### Validation of reduced m/z feature panels

The predictive strength of the selected m/z feature panels was further validated using Support Vector Machine (SVM) and Logistic Regression (LR) models applied to the top 25 features identified by both the VIP score and Gini index methods. As shown in Table 3, classification performance remained consistently high across both cohorts and selection approaches.

**Table 3:** Test set classification performance (20% hold-out) using Support Vector Machine (SVM) and Logistic Regression (LR) models based on the top 25 m/z features selected by Variable Importance in Projection (VIP) scores and Gini index in cohort A and cohort B (see Table 2)

Cohort	Feature selection	Model	Accuracy (%)	F1 score (%)
A	VIP	SVM	86	92
A	VIP	LR	90	94
A	Gini index	SVM	85	88
A	Gini index	LR	83	87
B	VIP	SVM	95	96
B	VIP	LR	87	91
B	Gini index	SVM	98	99
B	Gini index	LR	93	95

Notably, cohort B showed outstanding results, with Gini-based selection yielding a peak SVM accuracy of 98% and F1 score of 99%. The highest performance in cohort A was observed with VIP-selected features under LR, reaching 90% accuracy and 94% F1 score.

These results underscore the robustness and transferability of the selected m/z features across different classification models. The consistently high F1 scores indicate a strong balance between sensitivity and precision—an essential requirement in biomedical applications. Notably, the close agreement between VIP- and Gini-based selections highlights the methodological consistency of these feature selection strategies. While both approaches are effective, the RF-based Gini index offers added advantages in capturing complex, non-linear relationships, making it especially well-suited for metabolomic cohorts characterized by high dimensionality and limited sample sizes.

**Fig. 5:**
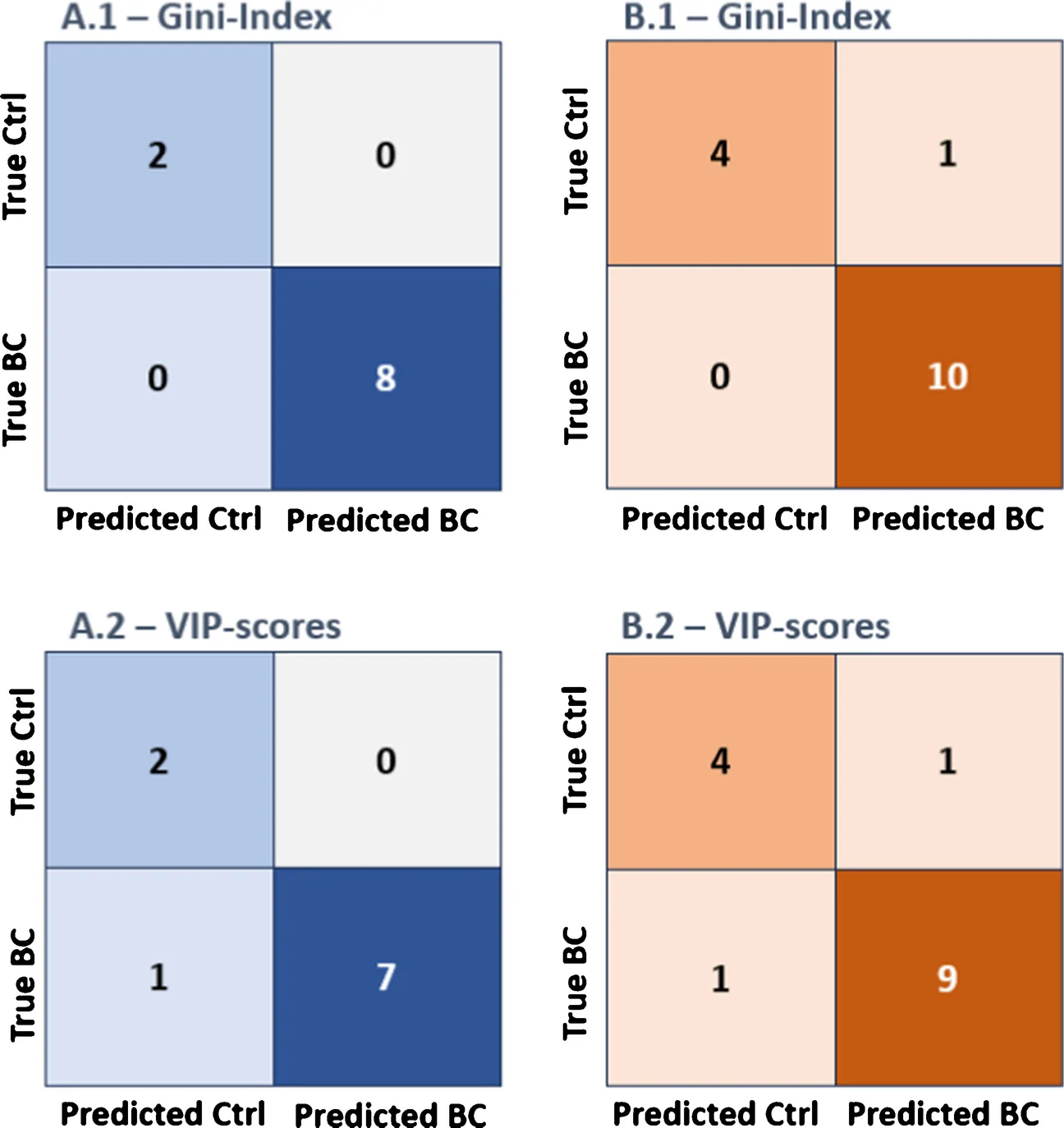
Mean confusion matrices for the reduced m/z feature panels from cohort A and cohort B selected using the Gini index (A.1, B.1) and VIP-scores (A.2, B.2). The Gini index-based panels exhibit stronger diagonal characteristics, indicating higher classification accuracy compared to the VIP-based counterparts

To further evaluate the classification performance of the top 25 m/z features selected via Gini index and VIP scoring, confusion matrices were generated for both cohort A and cohort B using the respective reduced panels (Fig. 5). The test sets consisted of 20% of the total samples, 10 samples for cohort A and 15 samples for cohort B. Notably, the Gini-based m/z feature panels demonstrated strong performance on these independent test sets. In cohort A, the model achieved a high average classification accuracy, while in cohort B, only a single false positive was observed, indicating highly accurate test set predictions.

These results closely resemble the performance observed with the full feature models (Fig. 2), highlighting the strong discriminative capability of the reduced m/z feature panels. Although the VIP-derived panels also achieved high classification accuracy, they exhibited slightly higher misclassification rates. This comparison indicates the enhanced predictive focus and robustness of the Gini-ranked m/z feature panels. Several limitations should be considered when interpreting these findings. Available cohort-level demographic information indicates an age difference between BC and Ctrl participants, particularly in cohort A. Because individual-level demographic variables were not available for all samples in the present retrospective analysis, the RF models could not be adjusted for age, BMI, menopausal status, or other clinical covariates. Therefore, demographic or physiological factors may have contributed to part of the observed metabolic separation. In addition, although the two cohorts originated from separate studies and were analyzed independently, patient-level identifiers were not available for retrospective verification of participant overlap. These limitations mainly affect the clinical interpretation of the selected panels. Accordingly, the reported m/z feature panels should be regarded as putative discriminatory feature sets for comparing feature-selection strategies, rather than as clinically validated breast cancer biomarkers.

## Conclusions

Overall, this leakage-aware and feature-preserving analytical framework provides a reproducible and transferable strategy for dimensionality reduction in untargeted metabolomics. By combining methodological rigor with interpretability, it strengthens the analytical foundation for metabolomics-driven biomarker discovery and future clinical validation.

This study demonstrates that supervised machine learning integrated with untargeted UHPLC–QTOF–ESI⁺ urinary metabolomics enables reproducible discrimination between breast cancer (BC) and control samples across two independent cohorts analyzed using distinct chromatographic workflows. Despite differences in feature dimensionality (224 vs. 129 aligned m/z features), Random Forest (RF) models achieved strong classification performance, with mean test accuracies of 86.7% in cohort A and 94.4% in cohort B. Receiver operating characteristic (ROC) analysis yielded a mean area under the ROC curve (ROC–AUC) of 0.96 in cohort A and values approaching unity in cohort B under strict leakage-controlled validation. Cohort B exhibited greater inter-split stability, likely reflecting its larger sample size, improved class balance, and clearer metabolic feature separation between classes.

A central strength of this work lies in the consistent application of the same RF-based analytical framework to two analytically distinct cohorts generated under different chromatographic separation conditions and comprising different metabolite feature spaces. Despite these analytical and dimensional differences, the identical algorithmic strategy-incorporating leakage-controlled training, Gini index–based feature ranking, and structured validation-yielded comparably high classification performance in both datasets. This cross-cohort consistency underscores the methodological robustness and transferability of the approach.

A further key contribution is the use of RF with embedded Gini index–based feature ranking for controlled dimensionality reduction. In contrast to projection-based approaches such as Principal Component Analysis (PCA) or Partial Least Squares Discriminant Analysis (PLS-DA), which transform original m/z features into latent variables and may obscure direct biochemical interpretability, RF preserves the identity of individual m/z features while capturing non-linear relationships and feature interactions. Importantly, stability was maintained after reduction to compact 25-m/z feature panels in each cohort. The preservation of strong accuracy, F1 scores, and ROC/precision–recall (PR) characteristics across both full and reduced models indicates that the discriminative signal captured by the RF approach is not cohort-specific nor dependent on a particular separation workflow, but rather reflects reproducible discriminative feature patterns associated with BC.

The robustness of feature selection was further supported by substantial overlap between RF-derived Gini rankings and Variable Importance in Projection (VIP) scores from PLS-DA analysis performed in MetaboAnalyst 5.0, confirming cross-framework consistency. Collectively, these findings highlight the advantage of a unified, leakage-aware, supervised tree-based strategy capable of delivering stable classification and interpretable dimensionality reduction across independent untargeted metabolomics platforms. By combining methodological rigor, transferability, and biochemical interpretability, this framework strengthens the analytical foundation for metabolomics-driven biomarker discovery and future clinical validation.

## Supplementary Information

Below is the link to the electronic supplementary material.

Supplementary file1 (DOCX 337 KB)

## Data Availability

The data that support the findings of this study are available from the corresponding authors upon reasonable request. Public Repository Python Code: https://github.com/MarkusZetes/metabolomics-dimensionality-reduction-script

## Declarations

### Source of biological material

Urine samples from Cohort A were collected from patients recruited at the Ion Chiricuță Oncologic Institute, Cluj-Napoca, Romania, and the Oncosurg Surgical Oncology Clinic, Cluj-Napoca, Romania. Urine samples from Cohort B were collected from patients at the Ion Chiricuță Oncologic Institute, Cluj-Napoca, Romania.
