# Supplementary material for: Dimensionality reduction and metabolite panel derivation in urinary metabolomics based on Random Forest with Gini index feature selection
Source: Anal Bioanal Chem. 2026 Sep 2;418(23):7889–902. doi: 10.1007/s00216-026-06759-w (PMC13744507; doi:10.1007/s00216-026-06759-w)
Supplement: Supplementary file 1 [file 216_2026_6759_MOESM1_ESM.docx]

Supplementary Information

for

**Dimensionality reduction and metabolite panel derivation in urinary metabolomics based on Random Forest with Gini index feature selection**

Markus Zetes^1^, Vlad Moisoiu^1^, Carmen Socaciu^2,3^, Nicolae Leopold^1*^

*^1^Faculty of Physics, Babeș-Bolyai University, 400084 Cluj-Napoca, Romania*

*^2^Faculty of Food Science and Technology, University of Agricultural Sciences and Veterinary Medicine, 400372 Cluj-Napoca, Romania*

*^3^BIODIATECH Research Centre for Applied Biotechnology in Diagnosis and Molecular Therapy, SC Proplanta SRL, 400478 Cluj-Napoca, Romania*

*Corresponding author Nicolae Leopold, Email: [nicolae.leopold@ubbcluj.ro](mailto:nicolae.leopold@ubbcluj.ro)

**Figure S1 and S2** provide a detailed analysis of the confusion matrixes behavior, confirming that both false-negative and false-positive cases are exceedingly rare across all test splits in cohort A and B. Each test split included a randomly selected subset of 10, respectively 15 independent urine metabolite samples.

**Figure S1.** Confusion matrices of the cohort A test set exhibit strong diagonal characteristics across all six splits, reinforcing the robustness of the model and its independence from the specific training split. (0 - control samples; 1 - breast cancer samples.) For details on training and test set sizes, refer to Section 2.4: Statistical and machine learning analysis.

**Figure S2.** Confusion matrices of cohort B test set exhibit strong diagonal characteristics across all six splits, reinforcing the robustness of the model and its independence from the specific training split. (0 - control samples; 1 - BC samples). For details on training and test set sizes, refer to *Section 2.2: Statistical and machine learning analysis*.

**Table S1.** m/z features (n = 25) ranked by their contribution to model performance, as quantified by the Gini index and Variable Importance in Projection (VIP) score for cohorts A and B. m/z features common to both rankings within each cohort dataset are highlighted in bold.

| **Importance rank** | **Dataset A m/z features** | **RF log GINI Index** | **Dataset A m/z features** | **VIP Score** | **Dataset B m/z features** | **RF log GINI Index** | **Dataset B m/z features** | **VIP Score** |
| --- | --- | --- | --- | --- | --- | --- | --- | --- |
| 1 | **457.319** | 0.04826 | **457.319** | 2.3543 | **360.326** | 0.07328 | **338.344** | 1.9946 |
| 2 | **391.333** | 0.04379 | **391.333** | 2.1278 | 343.299 | 0.06538 | **360.326** | 1.9667 |
| 3 | 644.506 | 0.04333 | **539.361** | 2.0104 | **371.33** | 0.05423 | **557.372** | 1.8851 |
| 4 | **539.361** | 0.04000 | **369.351** | 1.9968 | **557.372** | 0.05122 | **406.332** | 1.7843 |
| 5 | 688.533 | 0.03626 | **427.393** | 1.8764 | 129.128 | 0.04896 | **348.255** | 1.7311 |
| 6 | 161.971 | 0.03586 | **851.491** | 1.8736 | **338.344** | 0.04819 | **304.26** | 1.6939 |
| 7 | 184.987 | 0.03375 | **341.319** | 1.7726 | 228.27 | 0.04728 | 540.451 | 1.683 |
| 8 | **369.351** | 0.03373 | 230.251 | 1.6933 | **240.234** | 0.04530 | 280.265 | 1.6549 |
| 9 | 540.456 | 0.03243 | 440.362 | 1.6742 | 199.171 | 0.04187 | 277.186 | 1.6405 |
| 10 | **143.96** | 0.03072 | 663.466 | 1.6733 | **406.332** | 0.04151 | 545.407 | 1.6246 |
| 11 | 584.484 | 0.02877 | 282.282 | 1.6308 | 295.194 | 0.03968 | **350.269** | 1.6239 |
| 12 | 457.356 | 0.02875 | **426.472** | 1.6276 | 239.163 | 0.03665 | 496.425 | 1.5873 |
| 13 | 501.383 | 0.02793 | 327.081 | 1.6097 | 353.268 | 0.03429 | 501.38 | 1.5815 |
| 14 | 135.118 | 0.02750 | 612.175 | 1.6078 | **304.26** | 0.03233 | **393.312** | 1.5405 |
| 15 | 149.134 | 0.02583 | 412.456 | 1.5919 | **517.378** | 0.02450 | **371.33** | 1.5172 |
| 16 | **851.491** | 0.02293 | 256.302 | 1.5751 | 230.25 | 0.02233 | 285.28 | 1.507 |
| 17 | **427.393** | 0.02284 | 221.155 | 1.5716 | **348.255** | 0.02137 | 256.265 | 1.5031 |
| 18 | 172.956 | 0.02124 | 379.285 | 1.5707 | **393.312** | 0.02084 | 299.164 | 1.4574 |
| 19 | 413.328 | 0.01999 | 317.137 | 1.5631 | 173.152 | 0.01928 | **240.234** | 1.4492 |
| 20 | 496.428 | 0.01708 | 200.239 | 1.5609 | **531.261** | 0.01906 | **517.378** | 1.4469 |
| 21 | **145.956** | 0.01708 | **143.96** | 1.5594 | 200.222 | 0.01755 | 457.354 | 1.4358 |
| 22 | **341.319** | 0.01491 | 228.271 | 1.5218 | 214.255 | 0.01602 | 365.196 | 1.4275 |
| 23 | 207.111 | 0.01418 | **145.956** | 1.5166 | 288.274 | 0.01501 | **531.261** | 1.4105 |
| 24 | 429.323 | 0.01417 | 897.656 | 1.513 | **350.269** | 0.01068 | 282.281 | 1.4069 |
| 25 | **426.472** | 0.01231 | 459.493 | 1.5095 | 271.19 | 0.00965 | 227.166 | 1.3974 |

**Comparison of Feature Selection by Gini Index, VIP Scores, MetaboAnalyst Random Forest and Metabolic Feature Identification**

Using the MetaboAnalyst 5.0 platform, we extracted PLS-DA VIP scores (VIP > 1.5) and Random Forest (RF) scores (MDA > 0.003) for the top 25 metabolites in cohorts A and B. These results were compared with the Gini index–based feature ranking described in the main manuscript.

**Table S2** lists the top 25 metabolites identified by each method, while **Figure S3** illustrates the overlap of common *m/z* values across Gini, VIP, and MetaboAnalyst-RF rankings for both datasets.

Interestingly, no direct overlap was observed between the top metabolites of cohort A and cohort B, regardless of method. However, within each dataset, stronger overlaps emerged between the Gini index and MetaboAnalyst RF rankings, reflecting consistency between tree-based approaches. In contrast, fewer metabolites were shared between the Gini index and VIP methods, which is expected given their different statistical foundations (non-linear *vs.* linear projection).

Several methodological factors explain the discrepancies between cohorts A and B: (i) different extraction protocols, (ii) differences in separation and filtration procedures, and (iii) distinct *m/z* coverage (cohort A: 224 metabolites, 100–900 Da; cohort B: 129 metabolites, 100–600 Da).

Importantly, these differences do not indicate inconsistency or instability of the models, but rather highlight the context-dependent nature of metabolomic signatures. The results emphasize the necessity of validating candidate biomarkers across independent cohorts and analytical platforms. Moreover, they underscore the importance of harmonization and standardization in metabolomics workflows to ensure reproducibility and robustness of biomarker discovery.

**Table S2.** Top 25 metabolites ranked by their contribution to model performance, as quantified by the Gini index, VIP scores, and MetaboAnalyst-RF scores for cohorts A and B.

| Importance rank | **Cohort A** | | | **Cohort B** | | |
| --- | --- | --- | --- | --- | --- | --- |
|  | m/z features – Gini index | m/z features – VIP score | m/z features – RF score | m/z features – Gini index | m/z features – VIP score | m/z features – RF score |
| 1 | 457.319 | 457.319 | 425.220 | 360.326 | 338.344 | 338.344 |
| 2 | 391.333 | 391.333 | 457.319 | 343.299 | 360.326 | 360.326 |
| 3 | 644.506 | 539.361 | 688.533 | 371.33 | 557.372 | 304.260 |
| 4 | 539.361 | 369.351 | 319.287 | 557.372 | 406.332 | 371.330 |
| 5 | 688.533 | 427.393 | 440.362 | 129.128 | 348.255 | 557.372 |
| 6 | 161.971 | 851.491 | 501.383 | 338.344 | 304.260 | 343.299 |
| 7 | 184.987 | 341.319 | 363.314 | 228.27 | 540.451 | 240.234 |
| 8 | 369.351 | 230.251 | 369.351 | 240.234 | 280.265 | 406.332 |
| 9 | 540.456 | 440.362 | 391.333 | 199.171 | 277.186 | 353.268 |
| 10 | 143.96 | 663.466 | 327.081 | 406.332 | 545.407 | 348.255 |
| 11 | 584.484 | 282.282 | 413.328 | 295.194 | 350.269 | 227.166 |
| 12 | 457.356 | 426.472 | 539.361 | 239.163 | 496.425 | 517.378 |
| 13 | 501.383 | 327.081 | 329.163 | 353.268 | 501.380 | 545.407 |
| 14 | 135.118 | 612.175 | 379.285 | 304.26 | 393.312 | 501.380 |
| 15 | 149.134 | 412.456 | 221.155 | 517.378 | 371.330 | 199.171 |
| 16 | 851.491 | 256.302 | 402.361 | 230.25 | 285.280 | 285.280 |
| 17 | 427.393 | 221.155 | 457.356 | 348.255 | 256.265 | 167.034 |
| 18 | 172.956 | 379.285 | 279.162 | 393.312 | 299.164 | 393.312 |
| 19 | 413.328 | 317.137 | 524.460 | 173.152 | 240.234 | 239.163 |
| 20 | 496.428 | 200.239 | 443.339 | 531.261 | 517.378 | 457.354 |
| 21 | 145.956 | 143.960 | 584.484 | 200.222 | 457.354 | 540.451 |
| 22 | 341.319 | 228.271 | 349.063 | 214.255 | 365.196 | 531.261 |
| 23 | 207.111 | 145.956 | 380.341 | 288.274 | 531.261 | 277.186 |
| 24 | 429.323 | 897.656 | 325.238 | 350.269 | 282.281 | 332.334 |
| 25 | 426.472 | 459.493 | 256.302 | 271.19 | 227.166 | 326.381 |

**Figure S3.** Three-way Venn diagrams showing overlap among the top 25 m/z features selected by RF-Gini, PLS-DA VIP scores, and MetaboAnalyst RF rankings in (A) cohort A and (B) cohort B. m/z values were standardized to three decimal places before overlap calculation to avoid formatting-related mismatches. The central overlap represents m/z features selected by all three approaches.

Putative metabolite annotations for the most discriminative metabolic features identified in cohorts A and B are summarized in Tables S3 and S4. Metabolite assignment was performed based on accurate mass matching of the detected m/z values with entries from publicly available metabolomics databases, primarily the Human Metabolome Database (HMDB) and LipidMaps. The experimental precursor m/z values were compared with theoretical masses using a mass tolerance of 0.05 Da. These annotations should therefore be considered putative assignments based on MS¹ accurate mass matching, corresponding to MSI Level 3–4 identification confidence.

Notably, several of the annotated *m/z* features correspond to metabolites previously reported in metabolomics studies, including sulfolithocholic acid (*m/z* 457.319), palmitoleyl linolenate (*m/z* 501.383), phospholipid species (*m/z* 851.491), testosterone sulfate (*m/z* 369.351), ceramide species (*m/z* 540.456 and 584.484), 25-hydroxyvitamin D2 (*m/z* 413.328), and fatty acid derivatives (*m/z* 143.960). Their presence among the top-ranked features supports the biological plausibility and analytical relevance of the metabolic signatures observed in this study.

To complement the ROC-AUC values reported in the main manuscript, ROC curves (**Figure S4**) were generated from repeated held-out test predictions for both cohorts.

**Figure S4.** Receiver operating characteristic (ROC) curves for Random Forest classification in (A) cohort A and (B) cohort B. Thin grey lines represent individual held-out test splits, while the colored line represents the mean ROC curve. The shaded area in cohort A indicates ±1 standard deviation across splits. The dashed diagonal line represents random classification. The step-like profile reflects the limited number of samples in each held-out test partition. The near-perfect ROC-AUC observed in cohort B should be interpreted cautiously because of the moderate sample size and lack of prospective external validation.

**Table S3.** Putative metabolite annotations for the top-ranked metabolic features identified in **cohort A**. Annotations were assigned based on accurate mass matching of the detected *m/z* values with entries in HMDB and LipidMaps databases. Reported assignments correspond to putative annotations derived from MS¹ data

| **Rank** | **Cohort A Gini index ranking** | | | **Cohort A VIP score ranking** | | |
| --- | --- | --- | --- | --- | --- | --- |
|  | **m/z features – Gini index** | **Putative identification** | **ID (HMDB/ Lipidmaps)** | **m/z features – VIP score** | **Putative identification** | **ID (HMDB/ Lipidmaps)** |
| 1 | **457.319** | Sulfolithocholic acid | HMDB0000907 | **457.319** | Sulfolithocholic acid | HMDB0000907 |
| 2 | **391.333** | Ketodeoxycholic acid | HMDB0000328 | **391.333** | Ketodeoxycholic acid | HMDB0000328 |
| 3 | 644.506 | GlcCer(d14:1/16:0) | [LMSP0501AA39](https://www.lipidmaps.org/databases/lmsd/LMSP0501AA39) | **539.361** | FAHFA 16:0/5O(FA 18:0) | LMFA07090001 |
| 4 | **539.361** | FAHFA 16:0/5O(FA 18:0) | LMFA07090001 | **369.351** | Testosterone sulfate | HMDB0002833 |
| 5 | 688.533 | PE (16:1/16:1) | [LMGP02010108](https://www.lipidmaps.org/databases/lmsd/LMGP02010108) | **427.393** | Oleoylcarnitine | HMDB0005065 |
| 6 | 161.971 | Tryptamine | HMDB0000303 | **851.491** | TG(52:6) | [LMGL03014425](https://www.lipidmaps.org/databases/lmsd/LMGL03014425) |
| 7 | 184.987 | Thiouric acid | HMDB0245324 | **341.319** | Sulforaphane-N-acetylcysteine | HMDB0061113 |
| 8 | **369.351** | Testosterone sulfate | HMDB0002833 | 230.251 | Butenylcarnitine | HMDB0013126 |
| 9 | 540.456 | Cer(d18:0/16:0) | LMSP02020001 | 440.362 | Leukotriene E4 | HMDB0002200 |
| 10 | **143.96** | 5-Hydroxymethyluracil | HMDB0000469 | 663.466 | PA O-18:0/16:0 | [LMGP10020075](https://www.lipidmaps.org/databases/lmsd/LMGP10020075) |
| 11 | 584.484 | Cer(t18:0/18:0) | LMSP02030006 | 282.282 | 1-Methyladenosine | HMDB0003331 |
| 12 | 457.356 | Sulfolithocholic acid | HMDB0000907 | **426.472** | Elaidic carnitine | HMDB0006464 |
| 13 | 501.383 | Palmitoleyl linolenate | [LMFA07010121](https://www.lipidmaps.org/databases/lmsd/LMFA07010121) | 327.081 | Docosanol | HMDB0014770 |
| 14 | 135.118 | Malic acid | HMDB0000156 | 612.175 | Cer(t18:0/20:0) | [LMSP02030007](https://www.lipidmaps.org/databases/lmsd/LMSP02030007) |
| 15 | 149.134 | Mevalonic acid | HMDB0000227 | 412.456 | N-oleoyl glutamic acid | LMFA08020088 |
| 16 | **851.491** | TG(52:6) | [LMGL03014425](https://www.lipidmaps.org/databases/lmsd/LMGL03014425) | 256.302 | Sulfathiazole | HMDB0015619 |
| 17 | **427.393** | Oleoylcarnitine | HMDB0005065 | 221.155 | 5-Hydroxy-L-tryptophan | HMDB0000472 |
| 18 | 172.956 | Glycerol 3-phosphate | HMDB0000126 | 379.285 | Hydroxycortisol | HMDB0000418 |
| 19 | 413.328 | 25-Hydroxyvitamin D2 | HMDB0001438 | 317.137 | 4-Hydroxyretinoic acid | HMDB0006254 |
| 20 | 496.428 | LysoPC(16:0/0:0) | HMDB0010382 | 200.239 | 2-octenoylglycine | HMDB0094801 |
| 21 | **145.956** | N-Acetylcadaverine | HMDB0002284 | **143.96** | 5-Hydroxymethyluracil | HMDB0000469 |
| 22 | **341.319** | Sulforaphane-N-acetylcysteine | HMDB0061113 | 228.271 | Deoxycytidine | HMDB0000014 |
| 23 | 207.111 | Lipoic acid | HMDB0001451 | **145.956** | N-Acetylcadaverine | HMDB0002284 |
| 24 | 429.323 | Stearoylcarnitine | HMDB0000848 | 897.656 | TG(55:4) | [LMGL03010380](https://www.lipidmaps.org/databases/lmsd/LMGL03010380) |
| 25 | **426.472** | Elaidic carnitine | HMDB0006464 | 459.493 | LPA 20:4 | LMGP10050013 |

**Table S4.** Putative metabolite annotations for the top-ranked metabolic features identified in **cohort B**. Metabolite assignments were obtained through accurate mass matching with HMDB and LipidMaps databases. All annotations represent putative identifications based on MS¹ measurements

| **Rank** | **Cohort B Gini index ranking** | |  | **Cohort B VIP score ranking** | |  |
| --- | --- | --- | --- | --- | --- | --- |
|  | **m/z features – Gini index** | **Putative identification** | **ID (HMDB/ Lipidmaps** | **m/z features – VIP score** | **Putative identification** | **ID (HMDB/ Lipidmaps** |
| 1 | **360.326** | Epinephrine glucuronide | HMDB0010336 | **338.344** | 3-Indole carboxylic acid glucuronide | HMDB0013189 |
| 2 | 343.299 | D-Maltose | HMDB0000163 | **360.326** | Epinephrine glucuronide | HMDB0010336 |
| 3 | **371.33** | Androsterone sulfate | HMDB0002759 | **557.372** | PI (P-16:0/0:0) | [LMGP06070003](https://www.lipidmaps.org/databases/lmsd/LMGP06070003) |
| 4 | **557.372** | PI (P-16:0/0:0) | [LMGP06070003](https://www.lipidmaps.org/databases/lmsd/LMGP06070003) | **406.332** | Dehydrocholate | HMDB0304121 |
| 5 | 129.128 | Dihydrothymine | HMDB0000079 | **348.255** | Adenosine monophosphate | HMDB0000045 |
| 6 | **338.344** | 3-Indole carboxylic acid glucuronide | HMDB0013189 | **304.26** | Indoleacetyl glutamine | HMDB0013240 |
| 7 | 228.27 | Deoxycytidine | HMDB0000014 | 540.451 | Cer(d18:0/16:0) | LMSP02020001 |
| 8 | **240.234** | Carboxymethoxymethylguanine | HMDB0060805 | 280.265 | E-10-Hydroxynortriptyline | HMDB0013889 |
| 9 | 199.171 | Acetylamino-aminomethyluracil | HMDB0004400 | 277.186 | Glutarylcarnitine | HMDB0013130 |
| 10 | **406.332** | Dehydrocholate | HMDB0304121 | 545.407 | Estriol 3-sulfate 16-glucuronide | HMDB0010356 |
| 11 | 295.194 | gamma-Glutamylphenylalanine | HMDB0000594 | **350.269** | NAE 20:3 ( amide) | LMFA08040005 |
| 12 | 239.163 | Tyrosil glycine | HMDB0029105 | 496.425 | LysoPC(16:0/0:0) | HMDB0010382 |
| 13 | 353.268 | Prostaglandin E2 | HMDB0001220 | 501.38 | Palmitoleyl linolenate | [LMFA07010121](https://www.lipidmaps.org/databases/lmsd/LMFA07010121) |
| 14 | **304.26** | Indoleacetyl glutamine | HMDB0013240 | **393.312** | Androsterone sulfate | HMDB0002759 |
| 15 | **517.378** | MGMG(18:2/0:0) | [LMGL04010010](https://www.lipidmaps.org/databases/lmsd/LMGL04010010) | **371.33** | Androsterone sulfate | HMDB0002759 |
| 16 | 230.25 | Butenylcarnitine | HMDB0013126 | 285.28 | Thioinosinic acid | HMDB0060791 |
| 17 | **348.255** | Adenosine monophosphate | HMDB0000045 | 256.265 | Nicotinamide riboside | HMDB0000855 |
| 18 | **393.312** | Androsterone sulfate | HMDB0002759 | 299.164 | N-Acetylserotonin sulfate | HMDB0060834 |
| 19 | 173.152 | 2-Octenedioic acid | HMDB0000341 | **240.234** | Carboxymethoxymethylguanine | HMDB0060805 |
| 20 | **531.261** | Linolenyl stearate | [LMFA07010148](https://www.lipidmaps.org/databases/lmsd/LMFA07010148) | **517.378** | MGMG(18:2/0:0) | [LMGL04010010](https://www.lipidmaps.org/databases/lmsd/LMGL04010010) |
| 21 | 200.222 | 2-octenoylglycine | HMDB0094801 | 457.354 | Sulfolithocholic acid | HMDB0000907 |
| 22 | 214.255 | Indoxyl sulfate | HMDB0000682 | 365.196 | 6-Thioinosine-5'-monophosphate | HMDB0060416 |
| 23 | 288.274 | N-Ribosylhistidine | HMDB0002089 | **531.261** | Linolenyl stearate | [LMFA07010148](https://www.lipidmaps.org/databases/lmsd/LMFA07010148) |
| 24 | **350.269** | NAE 20:3 ( amide) | LMFA08040005 | 282.281 | 1-Methyladenosine | HMDB0003331 |
| 25 | 271.19 | Estrone | HMDB0000145 | 227.166 | Porphobilinogen | HMDB0000245 |
